# Taphonomic history and trophic interactions of an ammonoid fauna from the Upper Triassic Polzberg palaeobiota

**DOI:** 10.1038/s41598-022-11496-y

**Published:** 2022-05-06

**Authors:** Alexander Lukeneder, Petra Lukeneder

**Affiliations:** 1 Department of Geology and Palaeontology, Natural History Museum Vienna, Burgring 7, 1010 Vienna, Austria; 2 Vienna Doctoral School of Ecology and Evolution, University of Vienna, Djerassiplatz 1, 1030 Vienna, Austria

**Keywords:** Geology, Palaeontology, Sedimentology

## Abstract

The taphonomic mechanisms of a mono- to pauci-specific ammonoid fauna comprising 3565 specimens from the lower Carnian Polzberg *Konservat-Lagerstätte* near Lunz am See (Northern Calcareous Alps, Lower Austria) is described. The fossiliferous layers were deposited during the Julian 2 Ib (*Austrotrachyceras austriacum* Zone, *Austrotrachyceras minor* biohorizon). The deposits comprise abundant nektic ammonoids of the trachyceratid genus *Austrotrachyceras*. The bivalve *Halobia*, dominant among the invertebrates, is followed in abundance by the ammonoids *Austrotrachyceras* and *Paratrachyceras*, the coleoid *Phragmoteuthis* and frequent vertebrate actinopterygian fish. The monotonous ammonoid assemblage comprises abundant *Austrotrachyceras*, frequent *Paratrachyceras*, rare *Carnites* and *Simonyceras*. Recently collected ammonoids were sampled bed-by-bed and compared to extensive historical collections from the same localities. Bromalites (coprolites and regurgitalites) produced by large durophagous fish comprise ammonoid and fish masses and accompany the ammonoid-dominated Polzberg palaeobiota. The ammonoid fauna here presents a window into the nektic cephalopod world of the Upper Triassic assemblage and palaeoenvironment during the deposition of the fossiliferous layers. The frequent occurrence of the vertically oriented (external side horizontal to bedding plane) ammonoid shell fragments hint at a deposition after lethal fish or coleoid attacks. The Polzberg ammonoids were deposited under calm and dysoxic conditions in fine-laminated marlstones and shales of the lower Carnian Polzberg Sub-Basin within the Polzberg *Konservat-Lagerstätte*.

## Introduction

**Figure 1:**
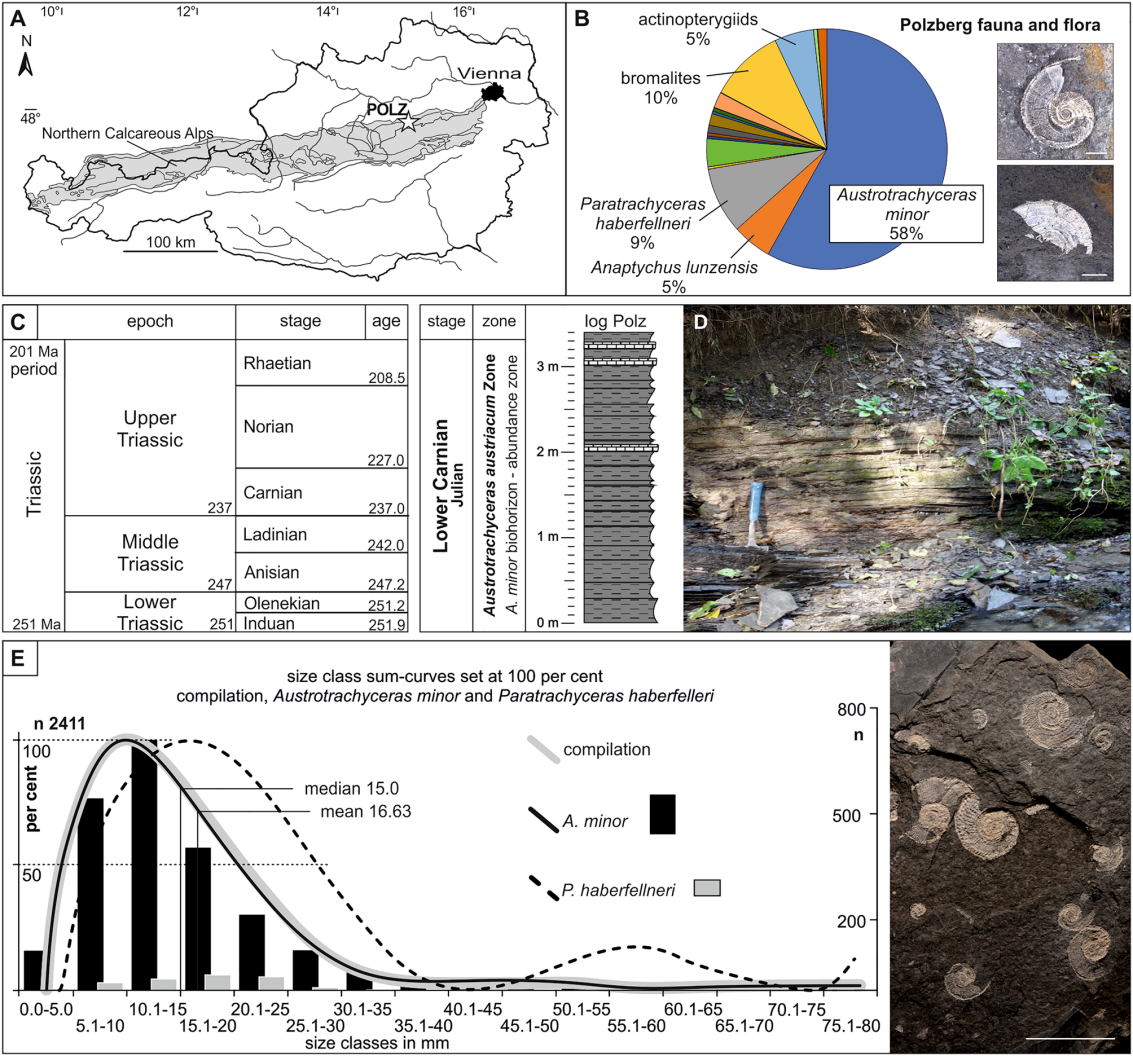
(**A**) Locality map of the Lunz area in Lower Austria and the Austrian Northern Calcareous Alps (in grey). Asterisk: position of the *Konservat-Lagerstätte* Polzberg (POLZ). (**B**) Pie chart of the Polzberg fauna and flora, indicated with dominant faunal elements, scale bars 1 cm. Images of specimens by AL. (**C**) Stratigraphic position of the Lower Carnian Polzberg palaeobiota. (**D**) with indicated detail of the section within the Carnian Reingraben Shales in the ravine Polzberggraben, 16 September 2021, by PL. (**E**) Compilation of size classes from n 2411 specimens of the genera *Austrotrachyceras* and *Paratrachyceras* and their species *A. minor* and *P. haberfellneri* with indicated example of ammonoid ontogenetic stages, image of specimens by AL, ammonoid layer in (E) NHMW 2021/0123/0139. Prepared by AL using CorelDRAW X7; www.coreldraw.com.

The Upper Triassic palaeobiota (*Austrotrachyceras austriacum* Zone) from the fossiliferous sites at Polzberg known as *Konservat-Lagerstätte*^1^ (see also^2,3^) yield a wealth of palaeobiological information^4–6^. Such marine conservation deposits provide unique insights into fossil assemblages and the taphonomic processes within their taxonomic groups^5,6^. No detailed report is available on the ammonoid taphonomy (biostratinomy and diagenesis) of the palaeobiota from the Polzberg area in the Northern Calcareous Alps. The Polzberg locality (= Schindelberg^7–9^ in historical collections) is situated in Lower Austria (Fig. 1A) and comprises the lower Carnian Reingraben Shales, which are fossiliferous in their basal few metres. Fossils from Polzberg are known since the nineteenth century^7–9^. More recently, new palaeontological data and faunal elements were revised and published from the Polzberg *Lagerstätte*^6^. The *Austrotrachyceras austriacum* acme Zone was deposited within the Upper Triassic Carnian Pluvial Episode (CPE^6,10–15^), a worldwide warming and humidification (enhanced rainfall) and characteristic Carnian carbonate platform crisis. During the Carnian (Late Triassic), the Polzberg area was located at the north-western rim of the Tethys in an area of 15° N to 30° N^10,12^. Environmental conditions changed during that episode. Subsequently, the inhabitants of the Triassic oceans and hence also in the Mediterranean Reifling Basin^6^ adapted to the prevailing special conditions. This is reflected in the composition of the ammonoid assemblage as well as in the distinct shell morphology and/or size reduction.

The present paper highlights new aspects in the Carnian ammonoid taphonomy^16^, from biostratinomy to diagenesis, and reports recent facts on the Polzberg ammonoid fauna (Fig. 1B) as well as on fossilized bromalites (regurgitalites and coprolites). The study presents the history and processes of the most prominent and abundant ammonoids (the trachyceratid genera *Austrotrachyceras* and *Paratrachyceras*) and concludes with hypotheses on reconstructed food webs including ammonoids from the Carnian *Konservat-Lagerstätte*. Thousands of historical and recently detected entire to fragmented ammonoid shells along with hundreds of bromalites provide new insights into the Upper Triassic (lower Carnian) taphonomic history of ammonoids^16–18^ and trophic aspects of the Polzberg palaeobiota.

### Geologic setting and lithology

The Upper Triassic outcrops at Polzberg (Polzberggraben ravine) are located on the western slope of Mount Schindelberg (1066 m), 4 km northeast of Lunz am See in Lower Austria (Lunz Nappe, Northern Calceraous Alps). The assignment of fossils and samples to the locality Schindelberg is synonymous with the locality Polzberg (= Pölzberg^6–8^; 1:50,000, geological map, sheet 71 Ybbsitz^19^, and sheet 72 Mariazell^20^; Fig. 1A). The exact position of the fossiliferous locality was determined by GPS (global positioning system): N 47° 53′ 4.98″ and E 15° 4′ 28.15″.

**Figure 2:**
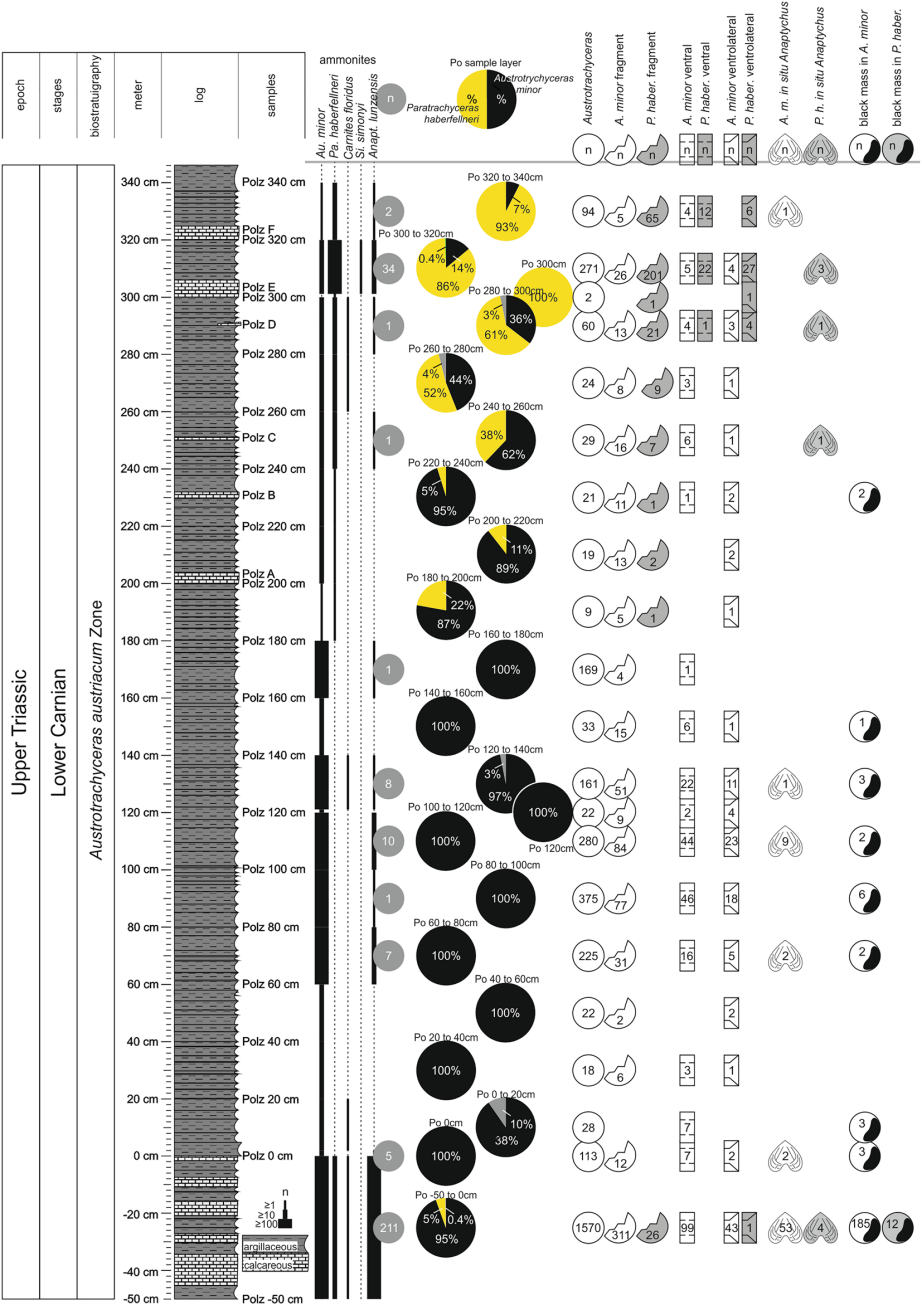
Detailed Polzberg section with indicated sampling layers (sample numbers Polz − 50 cm to Polz 340 cm) within the lower Carnian Reingraben Shales. Compilation of size classes from n 2411 specimens of the genera *Austrotrachyceras* and *Paratrachyceras* and their species *A. minor* and *P. haberfellneri* with indicated example of ammonoid ontogenetic stages, image of specimens by AL. Range of the occurring ammonoid taxa, with pie charts showing the percentage of *A. minor* (black) versus *P. haberfellneri* (yellow). From left to right: full circles—n of entire specimens of *Austrotrachyceras* and *Paratrachyceras*; open circles—n fragments of *A. minor* (white) and *P. haberfellneri* (grey); rectangles—ventral and ventrolateral position of *A. minor* (white) and *P. haberfellneri* (grey); in situ anaptychi and upper jaws in *A. minor* (white) and *P. haberfellneri* (grey); circle with black area—n specimens with black layer in *A. minor* (white) versus *P. haberfellneri* (grey). Prepared by AL using CorelDRAW X7; www.coreldraw.com.

Excavation campaigns to obtain the fossils were organised by the Geological Survey of Austria (GBA) in 1885^8,9^ and the Natural History Museum Vienna (NHMW) in 1909^6^. The historical, abandoned and collapsed mines were located at N 47° 53′ 23.31″ and E 15° 4′ 45.80″. More recently we sampled approx. 20 m downstream near the historical mine tunnels in the same fossiliferous layers (bed-by-bed). The basal part of the Reingraben Shales, directly above the Göstling Member (Fig. 1C, D), features a finely, distinctly millimetre-laminated *Ildefonso-type* interval (bright/dark stratification) without bioturbation^2,3,6^. This fossiliferous part bears abundant and unimodally distributed ammonoids (Fig. 1E) from the lowermost sample/ layer number Polz − 50 cm up to the topmost layer with Polz 320 cm in the section (Fig. 2). It contains the intercalated calcareous layers A to F. Pyrite is finely disseminated throughout the laminated, organic-rich marlstones and calcareous shales (CaCO_3_: 86.9% marly limestone to 2.9% in claystone/mudstone). Total Organic Carbon (TOC weight %) ranges from 0.3 to 1.4%, total sulphur (TS) from 0.3 to 1.8%^6^.

### The ammonoid taxa from the Polzberg *Konservat-Lagerstätte*

Thirty-seven fossil marine genera and land plant remains have been identified from the Polzberg palaeobiota. 6397 specimens (invertebrates and vertebrates) were reported from historical collections^6^, and additional 4953 fossil remnants were collected during five excavation campaigns in 2021. This yielded an enormous total of 11,350 fossil specimens. Around 1885 and 1909, thousands of ammonoids were collected from the Polzberg locality during the excavation campaigns of the GBA and the NHMW^8,9^. Stur^7^ and Teller^9^ were pioneers for this area and its Upper Triassic fauna and published preliminary data on the outcrops here. The palaeobiota shows a nekton-dominated fauna with abundant fish and cephalopods (ammonoids, coleoids)^5,6^. The main and abundant faunal elements are the flat clam species *Halobia rugosa* and the ceratitid *Austrotrachyceras minor*^2^ (= *Trachyceras triadicum* var. *minor*^21,22^ CLXXXVI = 186, p. 682).

The recent findings of 4953 fossils were the only ones collected bed-by-bed. Accordingly, the ecological, numerical and statistical investigations concentrated on that new fossil material, but incorporated the information hidden in the historical collections that were already compiled and analysed^6^. The faunal constituents are ammonoid conchs and anaptychi, coleoids (proostraca, phragmocones, hooks, cartilage), bivalves, gastropods, arthropods, polychaetes, conodontophorids, actinopterygians, chondrichtyes, dipnoids, coelacanths, bromalites (coprolites and regurgitalites) and plant remains. The cephalopod fauna is dominated by the ceratitid member of the Trachyceratidae, with *A. minor* comprising 86.7% (n 3077) and *P. haberfellneri* 13.2% (n 470); these two species represent 71.6% of the entire Polzberg palaeobiota. Entire ammonoids are present in size classes from 1 to 71 mm (Fig. 1E), frequently (n 57) with partly preserved buccal apparatuses of anaptychus-type lower jaws *Anaptychus lunzensis*^23^ and numerous upper jaws. Abundant *Austrotrachyceras* is accompanied by frequent *Paratrachyceras*, rare members such as *Carnites floridus* (n 17) and a single specimen of *Simonyceras simonyi* (n 1; Fig. 2). The lower jaw *A. lunzensis* was recorded with 281 isolated specimens throughout the section. The main historical Polzberg collections are housed at the NHMW and the GBA.

### Biostratigraphy: the *Austrotrachyceras minor* abundance zone

**Figure 3:**
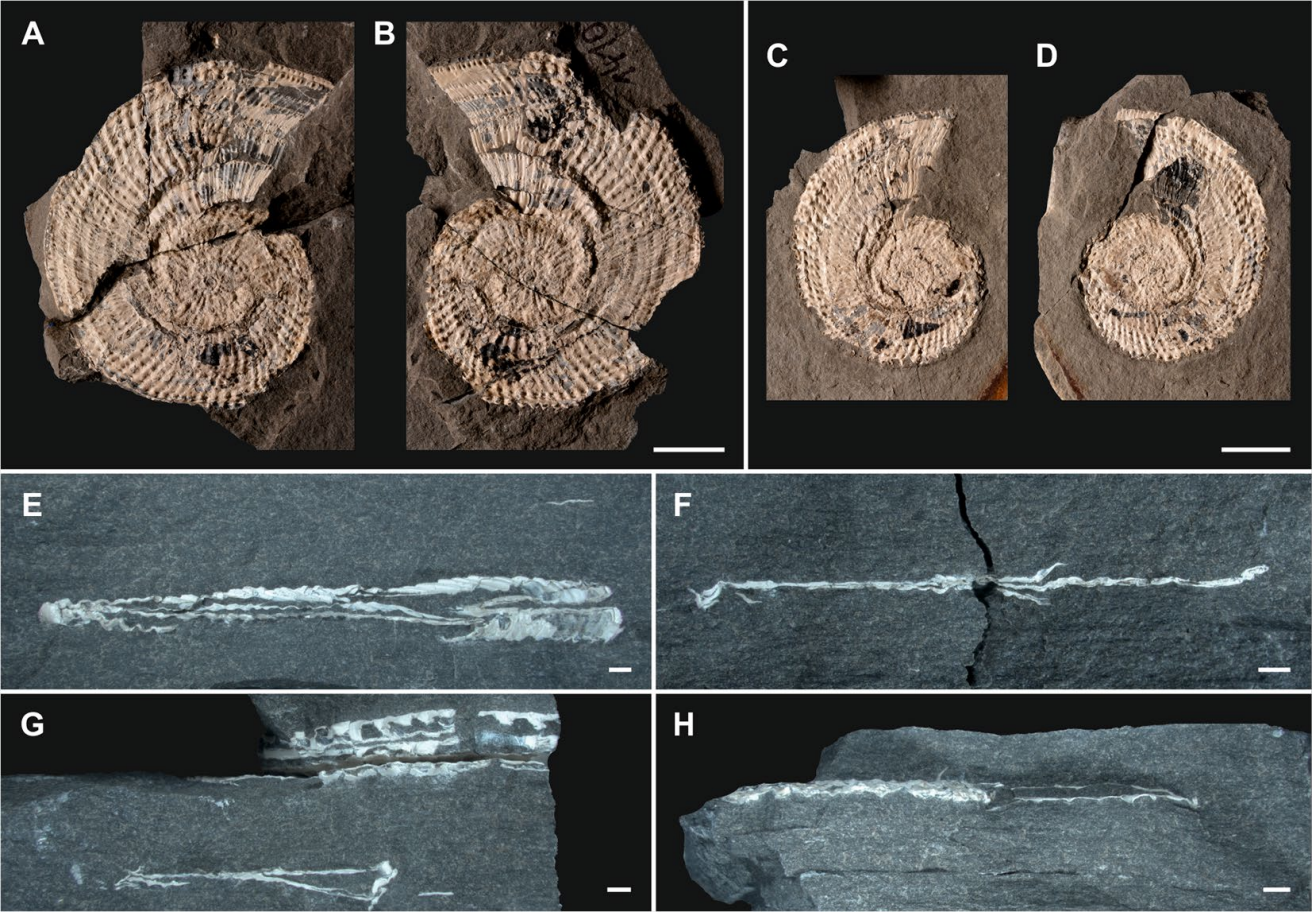
Ammonoid members of the Lower Carnian (Upper Triassic) Polzberg palaeobiota. (**A**) *Austrotrachyceras minor*, positive, lateral view, note change in adult shell ornamentation near aperture, NHMW 2021/0123/0131a; (**B**) *A. minor*, negative of (**A**), lateral view, NHMW 2021/0123/0131b; (**C**) *A. minor*, positive, lateral view, note change in adult shell ornamentation and black material near aperture representing the in situ *Anaptychus*, NHMW 2021/0123/0132a; (**D**) *A. minor*, negative of (C), lateral view, NHMW 2021/0123/0132b; (**E**) *A. minor*, crushed and compressed, horizontally embedded shell, sediment-filled body chamber, ventral view, NHMW 2021/0123/0133; (**F**) *A. minor*, crushed and compressed, horizontally embedded shell, body chamber, transect view, NHMW 2021/0123/0134; (**G**) *A. minor*, crushed and compressed, horizontally embedded shells, body chamber, top specimens ventral view on sediment-filled body chamber, lower specimen transect view, NHMW 2021/0123/0135a, NHMW 2021/0123/0135b; (**H**) *A. minor*, crushed and compressed, horizontally embedded shell, phragmocone (left) and sediment-filled body chamber (right), apertural view, NHMW 2021/0123/0136. Scale bars: (**A**–**D**) 10 mm, (**E**–**H**) scale bar: 1000 μm. Prepared by AL using CorelDRAW X7; www.coreldraw.com.

The lower Carnian fossiliferous deposits at Polzberg were deposited during the Julian 2 Ib (*Austrotrachyceras austriacum* Zone, *Austrotrachyceras minor* biohorizon; Figs. 1C, 2). The *Austrotrachyceras minor* biohorizon is underlain by the *A. triadicum* biohorizon and overlain by the *Neoprotrachyceras oedipus* Subzone with the basal *Austrotrachyceras* n. sp. 1 biohorizon^6,12,24^. The appearance of the abundant index ammonoids *A. minor* (Fig. 3A–D) within the fossiliferous interval (= abundance zones or “ammonoid beds”, characterized by abundance or mass-occurrence of ammonoids) is crucial for understanding the biostratigraphy of the lower Carnian (Julian) Polzberg *Konservat-Lagerstätte*.

### Preservation and taphonomy of the Polzberg ammonoid fauna

*Konservat-Lagerstätten* (stagnate environment), with their excellent preservation of fossils, form the special conditions required for the formation and conservation of entire and/or fragile and well-preserved fossil remains^1,4,6^. The preservation of benthic (epifaunal and infaunal) along with nektic taxa points to a deposition of the animals or their remnants within the palaeohabitat where the organisms primarily lived, swam and hunted, with no or minimal subsequent post-mortem drift or transport. No layers with densely packed ammonoid shells, accumulated by currents after catastrophic sudden-death events, are preserved or documented here. Conchs of nektic ammonoid (max. diameter from 1 to 77 mm; Figs. 1E, 2) and entirely preserved fish remains (max. length 22–334 mm) in the host rock exhibit no size sorting and lack orientation related to aperture or body axis by bottom water currents. Taphonomic evidence suggests that the Polzberg palaeobiota developed in oxygen-depleted basinal waters^6,25–27^, during calm conditions in the water column and near the sea floor, without major transport or reorientation of fossil material.

Well -preserved palaeocommunities of *Konservat-Lagerstätten* mirror the trophic conditions of the palaeo-food web at the time of deposition^1,4,6^: the shells or carcasses are not or only minimally affected by benthic scavengers or bacterial decay. Additional and frequent findings of bitten shell fragments (Fig. 2) crushed by nektic predators, along with numerous bromalites^28,29^ with coprolites and regurgitalites, shed light on the fossil record and the palaeobiota here^5,6^. Ammonoid shell fragments and entire shells are solely from the genus *Austrotrachyceras* with *A. minor* and *P. haberfellneri* (Fig. 1B, E); teuthid fragments stem exclusively from *Phragmoteuthis bisinuata*. Distinct coprolite masses are dominated either by fish scales, fragmented or entire ammonoid shells, coleoid (teuthids) hooks or carbonized cartilage remains. Specialised predators hunted for various kinds of prey and followed different predatory strategies. Actinopterygiid fish equipped with various dentitions for grinding and crushing fed on cephalopods or fish^5,6,30^. Near or at the sea floor, scavengers, grazers or decomposition of organic material by bacteria or fungi occurred.

#### Entire shells

**Figure 4:**
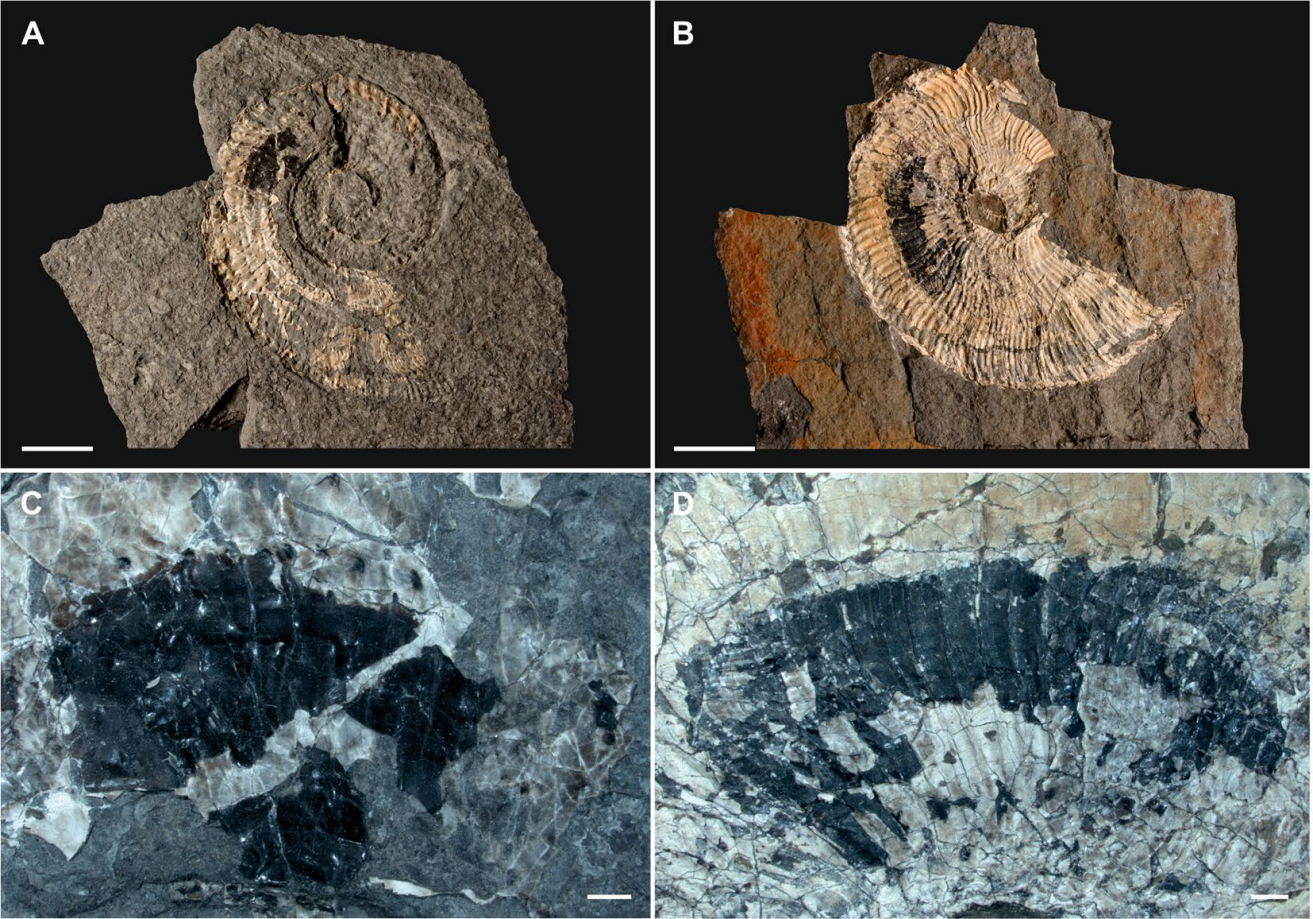
Ammonoid members and soft body remains of the Lower Carnian (Upper Triassic) Polzberg palaeobiota. (**A**) Adult *Austrotrachyceras minor*, positive, lateral view, note black mass in innermost posterior third of body chamber, NHMW 2021/0123/0137; (**B**) Adult *A. minor*, lateral view, with black mass in posterior third of body chamber, fatally bitten at ventral, NHMW 2021/0123/0138; (**C**) *A. minor*, magnification of black mass in (**A**), NHMW 2021/0123/0137; (**D**) *A. minor*, magnification of black area in (**B**), NHMW 2021/0123/0138. Scale bars: (**A**, **B**) 10 mm, (**C**, **D**) scale bar: 1000 μm. Prepared by AL using CorelDRAW X7; www.coreldraw.com.

Of the overall 3547 ammonoids collected, most shells (72.7%: n 2578) were entirely preserved and horizontally embedded (Figs. 2, 3E–H). Ammonoid shells are whitish, and scanning electron microscopy (SEM) and energy dispersive spectroscopy (EDS) analyses confirm that the shells preserve the original pristine aragonitic tablets. The shell walls in *Austrotrachyceras* and Paratrachyceras are composed of three distinct main layers, an outer prismatic layer, the main and thickest nacreous layer (columnar nacre) and an inner prismatic layer^31,32^. The inner prismatic layer is developed only in the posterior part of the body chamber; subsequently, the nacreous layer covers two-thirds of the anterior part to the aperture of the inside body chamber. In general, *Austrotrachyceras* is a small-sized, strongly ornamented trachyceratid ammonoid measuring up to 77 mm in diameter (Fig. 1E). During fossilisation, the shell material is partly transformed into calcite in a few distinct layers, depending on the primary lithological composition (i.e. argillaceous versus calcareous). The ammonoid shells are strongly compacted and their width reduced to 1–2 mm by diagenetic processes. The compaction caused fracturing of the outer shell as well as septal wall breakage (Fig. 3). The suture is visible in only a few specimens in which the external wall was extracted by sampling or preparation^33^. Shell size is not affected by compaction, as confirmed by the ventrally oriented preserved shells. External shell walls resisted the diagenetic pressure, are not crushed and hence preserved as an elevated ventral spire and visible on bedding planes. *Austrotrachyceras* specimens are often preserved with a black mass in the body chamber (Fig. 4), with frequently black (carbonized) anaptychi (*Anaptychus lunzensis*) in front of the body chamber aperture, a normal distribution through the section, a larger size in the lower part of the section, and an increase of the *Paratrachyceras haberfellneri* (n 470; 13%) ratio versus *Austrotrachyceras minor* (n 3077, 87%). Sporadic shell accumulations were recorded in particular distinct layers (n > 10 per dm^2^). All shell classes from juvenile, mid-aged to adults are present, mostly in the same layers. In *A. minor*, 77.3% (n 2378) of the conchs are complete, whereas in *P. haberfellneri* this value is 42.6% (n 200). All ontogenetic stages (juveniles to adult specimens) are present, showing a unimodal peak at the 10–15 mm size class (mean 15.4 mm, median 13.0 in *A. minor*; mean 16.9 mm, median 17.5 in *P. haberfellneri*) (Fig. 1E). The data (diameters) of all complete specimens measured (n 2378) show a unimodal, asymmetrical, positively right skewed distribution pattern (skewness 1.579; leptokurtic kurtosis 3.909) of size classes (Fig. 1E). To better compare curve shapes and visualise size classes, the curves were set to 100%. *A. minor* dominates within the interval from Po -50 cm up to Po 280 cm. From Po 280 on upward, *A. minor* is gradually replaced by the smoother *P. haberfellneri* (Fig. 2). *A. minor* has diameters ranging up to 77 mm (mean 16.6 mm) with its maximum in layer Po 0–20 cm and the minima in layers Po -50–0 cm and Po 300–320 cm. The smaller *P. haberfellneri* exhibits a diameter range of 3–33 mm (mean 16.9 mm) with its maximum in layer Po -50–0 cm and minimum in layers Po 320–340 cm.

#### Ammonoids with black masses

216 ammonoids exhibit an elongated area showing a black mass at the posterior end of the body chamber near the final septa, approx. a half whorl distance from the aperture. That layer starts near the umbilical edge, reaches up to half of the lateral wall, and does not extend to the venter/external side. The extension varies from 2 to 4 cm depending on the ammonoid ontogenetic stage. The black mass is approx. 1 mm thick, squeezed between lateral shell walls (Fig. 4A–D). The black material is amorphic, opaque, shiny black, brittle-breaking, partly with intercalated pockets filled by granular substance. EDS and SEM analyses show that the black substance consists almost exclusively of carbon (C). From Po − 50 cm up to Po 340 cm, 6.6% (n 204) of *A. minor* specimens exhibit such a black area within the body chamber. In the same interval, 2.6% (n 12) of the *P. haberfellneri* specimens exhibit a black layer.

#### Ammonoids with in situ jaws

**Figure 5:**
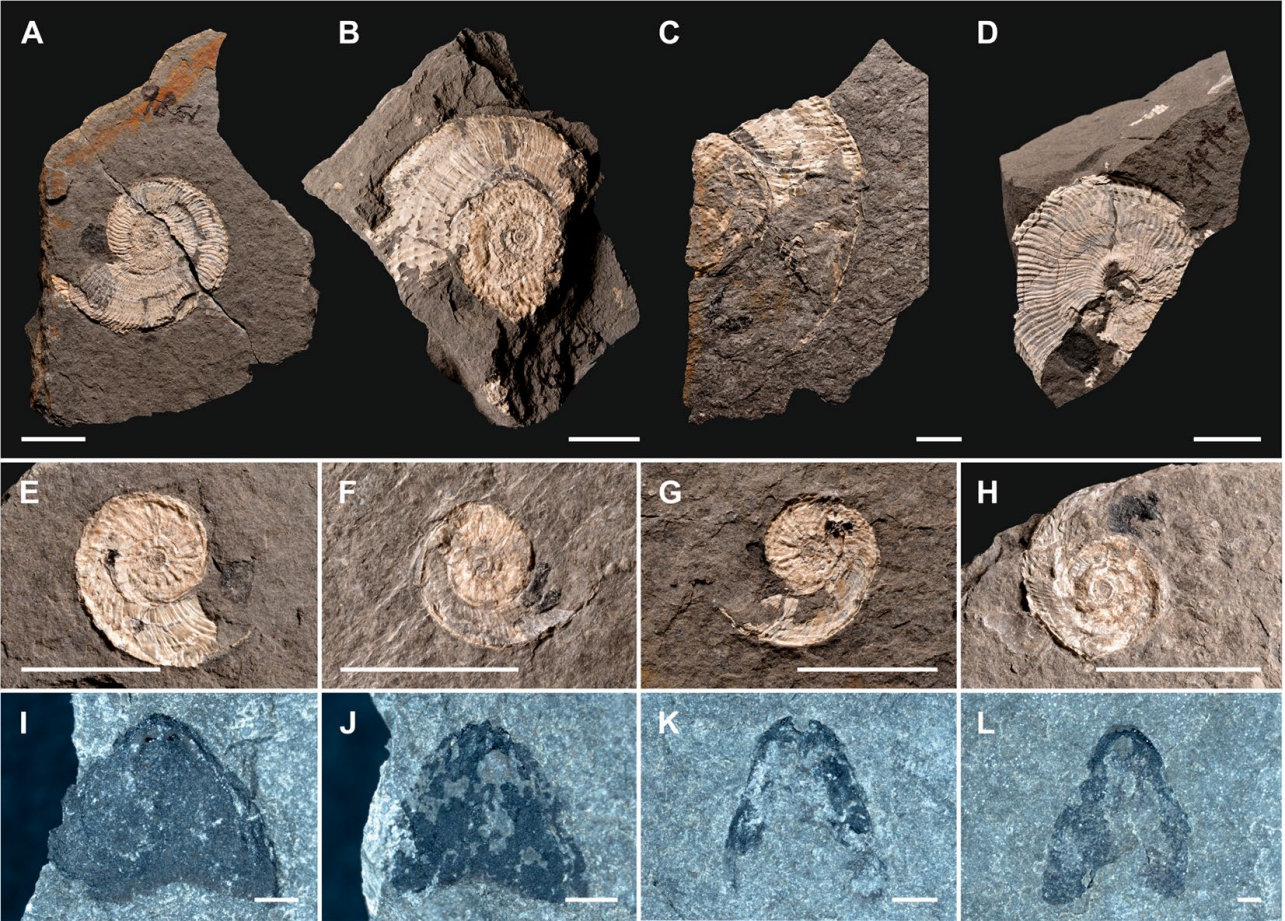
Ammonoid members and in situ anaptychi of the Lower Carnian (Upper Triassic) Polzberg palaeobiota. (**A**) *A. minor*, positive, lateral view, adult specimen with black *Anaptychus lunzenis* in front of aperture, NHMW 2021/0123/0141; (**B**) *A. minor*, lateral view, adult with black *A. lunzenis* in front of aperture, NHMW 2021/0123/0142; (**C**) *A. minor*, partly preserved positive, lateral view, adult with black *A. lunzenis* in anterior part of body chamber, NHMW 2021/0123/0143; (**D**) *P. haberfellneri*, lateral view, adult with black *A. lunzenis* in front of body chamber, NHMW 2021/0123/0144; (**E**) *A. minor*, positive, lateral view, juvenile with black *A. lunzenis* in front of body chamber, NHMW 2021/0123/0145; (**F**) *A. minor*, positive, lateral view, juvenile with black *A. lunzenis* in front of body chamber, NHMW 2021/0123/0146; (**G**) *A. minor*, positive, lateral view, juvenile with black *A. lunzenis* in front of body chamber, NHMW 2021/0123/0147; (**H**) *A. minor*, positive, lateral view, juvenile with black *A. lunzenis* in front of body chamber, NHMW 2021/0123/0148; (**I**) *A. lunzensis*, lower jaw, positive, lateral view, isolated specimen, NHMW 2021/0123/0149; (**J**) *A. lunzensis*, lower jaw, positive, lateral view, isolated specimen, NHMW 2021/0123/0150; (**K**) upper jaw, positive, lateral view, isolated specimen, NHMW 2021/0123/0151; (**L**) upper jaw, positive, lateral view, isolated specimen, NHMW 2021/0123/0152. Scale bars: (**A**–**H**) 10 mm, (**I**–**L**) scale bar: 1000 μm. Prepared by AL using CorelDRAW X7; www.coreldraw.com.

Ammonoid specimens (n 57) are often preserved with in situ jaws within or in front of the body chamber (Fig. 5). A total of 281 anaptychi were detected in the Polzberg section. These anaptychi represent lower jaws in buccal masses of trachyceratid ammonoids^23^. Anaptychi are rarely reported from pre-Jurassic deposits because they are primarily chitinous; when present, they are preserved as black, univalve plates. Analyses show that the black substance consists almost exclusively of enriched carbon (C) altered from a chitinous substance by carbonization in early diagenetic stages. From Po − 50 cm up to Po 340 cm (Fig. 5A–C), 1.75% (n 54; at the base 3.6%; Fig. 5E–H) of the *A. minor* specimens exhibit in situ anaptychi in the innermost third of the body chamber. The corresponding value for *P. haberfellneri* (Fig. 5D) in the same interval is 1.9% (n 12). 224 anaptychi of *A. lunzensis forma typica*^23^ (Fig. 5D, J) and *A. lunzensis forma longa*^23^ (Fig. 5K, L) were detected isolated from ammonoid shells on the bedding planes. The elongated morphotypes shown in Fig. 5K, L are in fact upper jaws of *Austrotrachyceras* and *Paratrachyceras*.

#### Fragmented shells

**Figure 6:**
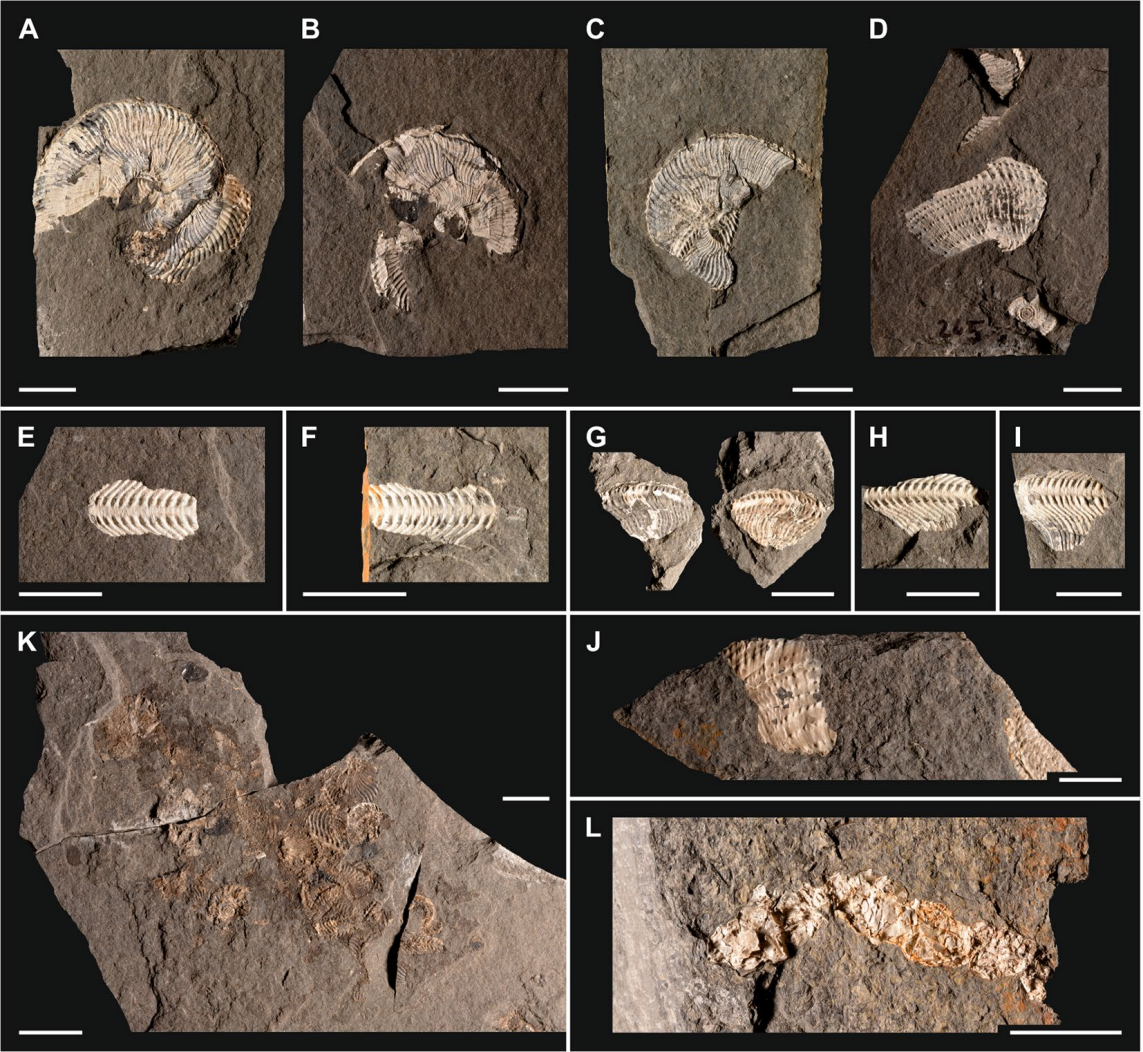
Ammonoid members with fatally bitten shells, fragments and bromalites of the Lower Carnian (Upper Triassic) Polzberg palaeobiota. (**A**) *P. haberfellneri*, positive, lateral view, adult specimens, fatally bitten ammonoid with dislocated shell fragments, NHMW 2021/0123/0153; (**B**) *P. haberfellneri*, positive, lateral view, adult specimens, fatally bitten ammonoid with dislocated shell fragments, NHMW 2021/0123/0154; (**C**) *A. minor*, fatally bitten ammonoid with dislocated shell fragments, NHMW 2021/0123/0155; (**D**) *A. minor*, lateral view, body chamber fragment, NHMW 2021/0123/0156; (**E**) *A. minor*, ventral view, ventrally oriented body chamber fragment, NHMW 2021/0123/0157; (**F**) *A. minor*, ventral view, ventrally oriented body chamber fragment, NHMW 2021/0123/0158; (**G**) *A. minor*, ventral view, ventrolaterally oriented body chamber fragment, NHMW 2021/0123/0159a, b; (**H**) *A. minor*, ventral view, ventrolaterally oriented body chamber fragment, NHMW 2021/0123/0160; (**I**) *A. minor*, ventral view, ventrolaterally oriented body chamber fragment, NHMW 2021/0123/0161; (**J**) *A. minor*, ventral view, ventrolaterally oriented body chamber fragment, NHMW 2021/0123/0162; (**K**) Bromalite mass with *A. minor*, *P. haberfellneri* and *Anaptychus lunzensis,* top view, entire and fragmented shells, NHMW 2021/0123/0163; (**L**) Bromalite chain, coprolite, consisting solely of ammonoid fragments, NHMW 2021/0123/0164. Scale bars: (**A**–**L**) 10 mm. Prepared by AL using CorelDRAW X7; www.coreldraw.com.

A high percentage (22.7%; n 1033) of the detected ammonoid shells are fragmented (Fig. 6). Fragment sizes range from three-fourths of the shell down to small pieces measuring only 2–3 mm (Fig. 6A–D, J). Fragmented shells and shell hash appear in the same layers as entire shells. Shell fragments have sharp edges and occur isolated or scattered on bedding planes with a dominating horizontal (parallel to bedding plane) orientation. Convex shell fragments are typically oriented in a stable hydrodynamic position with the convex side up. Throughout the section from Po − 50 cm up to Po 340 cm, 22.7% of the *A. minor* shells are fragmented. The corresponding value for *P. haberfellneri* is a very high 71.1%. *A. minor* fragment diameters range from 1 to 45 mm (mean 9.3 mm, median 8.0 mm). Fragment diameters of the smaller *P. haberfellneri* range from 1 to 33 mm (mean 7.7 mm, median 7 mm). The fragment size maximum in both species is in the size class from 5 to 10 mm.

#### Vertically oriented ammonoid shells

Compared to other ammonoid occurrences in laminated shale deposits, vertically oriented ammonoid shells (ventral or ventrolateral view; Fig. 6) are frequent: the ventral shell wall (= external side, n 304) or ventrolateral (n 165) shell edge is visible in horizontal position, with the original shell and sharp edges preserved. That special preservation lacks any traces of ammonoid imprints or roll marks—these are real shell fragments. *A. minor* exhibits 8.7% (n 269) of shells in ventral (Fig. 6E, F) and 4.0% (n 124) in ventrolateral orientation from Po − 50 cm up to Po 340 cm (Fig. 6G–I). In the same interval, the corresponding values for *P. haberfellneri* are 7.5% (n 35) in ventral and 8.7% (n 41) in ventrolateral orientation.

#### Ammonoids as constituents of bromalites

Bromalite^28,29^ masses (n 52) composed of variously oriented complete ammonoid specimens (with original white shell, with black layer and suture fragments), ammonoid hash with angular shell margins and teuthid cartilage fragments were detected^5,6^. The evidence suggests that the Polzberg locality preserves three types of bromalites, with coprolites and regurgitalites^34^ being the most common. Three main types occur. Type A represents large flattened (max. 5 mm thick), partly phosphatised masses with variously oriented complete or fragmented ammonoids (n 33, d 77 mm, nr 1664, Fig. 6K); n 22, d 82 mm nr 4034; type A in Lukeneder et al.^5^). These are assumed to be regurgitalites or oral ejecta. Type A masses appear with characteristic features for regurgitalites, namely thin, with randomly grouped and mixed angular shell fragments of different size, shell edges with low degree of roundness not significantly affected by gastric etching, and a lack of phosphatic matrix^5^. Type B (nr 2461; d 52 mm and 10 mm thick) encompasses rounded to sub-rounded, more massive and three-dimensionally preserved elements including more crushed, fragmented ammonoids and teuthid cartilage (n > 50, see type A Fig. 4A in Lukeneder et al.^5^). Type C presents smaller flattened areas comprising ammonoid shell fragments and ammonoid hash; these areas are cloud- or string-shaped and up to 30 mm long and 6 mm wide (Fig. 6L). More than 100–200 fragments occur (0.01–20 mm) in such coprolite strings and exhibit visible features of the corresponding taxa such as nodes and septal fragments (Fig. 6L). The same size classes are also visible in bitten ammonoid fragments preserved in small (3–11 mm, often from one ammonoid only) cloudy areas. Complete juvenile ammonoid conchs measuring 1.0 mm are rare. This material is accompanied by anaptychi and is often embedded in black organic material. An interesting feature within some regurgitalites is the presence of uncrushed ammonoid shells. Ammonoid shell fragments and entire shells are exclusively from the genus *Austrotrachyceras* (*A. minor* and *P. haberfellneri*, accompanied by anaptychi). All size classes from juveniles to adults were documented in the bromalites, entirely preserved or as fragments to shell hash. In other accumulations, ammonoid fragments predominate.

### Taphonomic history and trophic interactions of the Polzberg ammonoid fauna

Taphonomic mechanisms produce distinct and characteristic preservational features of ammonoid shells^16–18^. The relevant processes start with the death of the ammonoid, continue with the burial in the sediment (biostratinomy) and usually end with the period after burial with the diagenetic modification^16,17,35^. Biostratinomy involves biological processes such as decay or scavenging along with physical effects such as shell breakage or transport. These mechanisms end with the burial, followed by chemical processes that modify shell morphology or change its mineralogy^18^.

The spectrum of the mostly completely preserved ammonoids includes all ontogenetic stages from hatchlings to adults. In the taphonomic context, the ammonoid assemblage mirrors an autochthonous community without redeposition or transport out of habitat. The Polzberg ammonoid occurrence depicts a well-preserved, complete community of an autochthonous thanatocoenosis deposited in the Lower Carnian Polzberg sub-basin^6^. Processes such as scavenging, decay, disarticulation, abrasion, bioerosion, and corrosion were hindered or missing. This reflects the special environmental circumstances near and at the sea floor in the constricted basin^5,6^.

Doguzhaeva et al.^32^ reported bituminous soft body tissues in *Austrotrachyceras* (n 6) from historical specimens here. The authors assumed that this black substance was a mixture of lamellar and a subfibrous archaic mantle and ink substance, reworked by carbon-accumulating bacteria^32^. There is broad doubt in the cephalopod community that the described substance from the Polzberg trachyceratids represents primary ink^36^. As noted and confirmed by frequent own observations (SEM, EDS), the black material contains numerous taxa of spherical bacterial colonies and filamentous fungal structures. A hypothetical starting point to formulate ideas on the nature of that black substance are 1—the consistent posterior position in the body chambers, 2—the comparable oval outline and shape in all specimens, and 3—the composition and microstructure of the black substance. The idea of ink in *Austrotrachyceras*^32^ was refuted and attributed to measurement failures, most probably based on analyses of the black layer^36^ (pers. comm. C. Klug 2022). Triassic and Jurassic ammonoids exhibit the so-called black layer (“schwarze Schicht”^36,37^). Hypothetically, the ink sac evolved after the cephalopods positioned the shell into the soft body and took up an endocochleate mode of life^36,38^.

The area and outline of the black mass correlates with similar lateral attachment scars of muscle for the hyponome retractor in Jurassic ammonoids from Russia^39^, see^40^. Similar structures were described as ventrolateral muscle scars in Cretaceous *Aconeceras* from Russia^41^. New details are now available based on 3D neutron tomography and X-ray tomography of the position of mantle and muscle soft parts in Middle Jurassic ammonoids^42^. The positions of cephalic retractor and hyponome retractor muscles positions correlate with the black area described from the austrotrachyceratid specimens shown herein.

*Anaptychus lunzensis*^23^ was described from the Polzberg deposits as being the lower jaws of *Paratrachyceras haberfellneri* (= “*Trachyceras haferfelneri forma typica*”^23^). Trauth^23^ was uncertain in other form types such as *forma longa* (upper jaws, Fig. 5K, L), *forma lata* and *forma carinifera* in *Anaptychus lunzensis*. He mentioned a possible connection to *Trachyceras triadicum* and *Trachyceras austriacum*, which are now considered as being synonyms of *Austrotrachyceras minor*. Note here that Trauth^23^ assigned the anaptychi as having an operculum function in the corresponding ammonoid taxa. Anaptychi located in the aperture, thus suggesting a potential operculum-function, are also reported from in situ findings of Devonian ammonoids^43^. Other buccal elements from the Moroccan Devonian lie in the body chamber and are interpreted as mouth parts^44^. We assume that the different forms depict lower (Fig. 5I, J) and upper jaws (Fig. 5K, L) of the described ammonoid taxa *A. minor* and *P. haberfellneri.*

Biostratinomically, entirely preserved ammonoids exhibiting in situ buccal masses are interpreted as quasi autochthonous faunal elements: the intact shells sank after death of the animal and neither surfaced nor drifted far from their original habitats^16,18,43,45^. Many pre-Jurassic ammonoids had non-mineralized jaws similar to modern coleoid beaks^46^. Assigning fossil buccal masses to their respective species involves diverse palaeoecological and taphonomic features in ammonoid science. The exact correlation of isolated jaw elements has been solved for numerous ammonoid taxa^47^. The anaptychi described herein are interpreted as non-mineralized trachyceratid lower and upper jaws (Fig. 5I, J vs 5 K, L; pers. comm. K. Tanabe 2022). The taphonomic position of the preserved in situ jaws suggests rapid deposition after death. Rather than being drifted, the animals became rapidly waterlogged and sank to the sea floor with intact buccal masses^48^. This scenario is also strengthened by the other faunal data (entire fish carcasses, well-preserved bristle worms), the sedimentological (lamination, no bioturbation, black) and the geochemical data (total organic carbon, pyrite). The cephalopod versus aptychi ratio (even in situ) is a useful criterion for evaluating the postmortem transport of ammonoids^18,48–52^.

Shell fragments bear sharp edges caused by bite attacks of fish or coleoids. No marine reptiles have been found here^6^ and are therefore currently excluded as possible predators of ammonoids. The vertically and subvertically preserved shell remains from Polzberg are exclusively fragments. This contrasts with the data given for the Cretaceous (Campanian) example from Antarctica and points to a different palaeoenvironment^53^. Various types of vertically embedded ammonoid shells were probably primarily oriented by the rapid sedimentation within dense suspensions during the Cretaceous in Antarctica^53^. No differentiation in taphonomic behaviour between different morphotypes is evident in those environments and the vertical deposition occurred in all taxa. The presence of landing or touch marks near some of the vertically preserved ammonoids^53^ demonstrate vertical sinking^16,18,52,53^ and thus explain the rare vertical position and preservation^54–56^. Nonetheless, the normal preservation is with the plane of symmetry oriented parallel to the bedding.

Most of the Polzberg ammonoid shell fragments were isolated and separated from the rest of the shell on the bedding planes. This is interpreted as deposition after the animal was bitten and the conch crushed within the water column, with the fragments sinking subsequently down to the sea floor without much drifting or redeposition. Vertical orientation occurs in all the Polzberg ammonoid morphotypes in all facies and lithology types (argillaceous intervals with dolomitic limestone layers). The analyses of the shell fragments indicate no postburial reworking because bioturbation is absent in the laminated sediments, reflecting a dysoxic to anoxic substrate.

Shell damage is a powerful indicator for predator–prey interactions^18,57–59^ involving different predator groups in the Late Triassic palaeobiota of Polzberg. Ammonoids, mainly the dominant genus *Austrotrachyceras* with *A. minor* and *P. haberfellneri*, are both predators and prey, foraging and being attacked in the same habitat. Sublethal and lethal shell damage from recent and fossil cephalopods are well known^18,60,61^. Predation in the water column (exact water depth not specified) may have involved ichthyosaurs, mosasaurs, nothosaurs, sharks or other fishes (semionitids^59^, pycnodonts^62,63^, holostei^64^, teleosts^65^), and invertebrates including other ammonoids, nautiloids and coleoids^18,59,61,66^. Modern cephalopods (squids, cuttlefish, octopuses) are amongst prey for predatory fish worldwide^67^. Live attacks by the modern actinopterygiid fish *Dentex* (Sparidae) have for example been observed on modern *Sepia* cuttlebones from the Atlantic (own observations A. Lukeneder). As documented for a variety of Mesozoic ammonoid groups^68,69^ and for Jurassic ventral bite marks^70,71^ from Lyme Regis in the UK, most attacks were probably caused by teuthids actively preying on the living ammonoid animals^18,61^. The Polzberg palaeobiota supports this interpretation: numerous specimens are preserved with ventrally and fatally bitten shells. Fish generally broke parts off from the aperture to expose the soft body, as observed in modern *Nautilus* attacked by parrotfish^72^, triggerfish and groupers^60^. Teuthids, in turn, attacked the ventral parts of the ammonoid shells. Injuries inflicted by extant fishes can be sublethal or lethal, in contrast to the fatal bite attacks by coleoid members indicated by the fossil evidence. Clusters of broken ammonoid shells^66^ are frequent in the Polzberg palaeobiota.

Marine actinopterygiids make up 55% of predatory genera within the fish community at Polzberg^5,6,14^, with the largest predatory member being *Saurichthys*. This active predator hunted other actinopterygiid fish^73^ and probably also small trachyceratid ammonoids. No sublethal injuries are reported on ammonoid or coleoid specimens here—only fatally bitten and crushed or fragmented cephalopods—pointing to immediate death by predators specialized on nektic cephalopods. In contrast, the coleoid *Phragmoteuthis* could have fed on actinopteyigiid fishes and hunted small and slow austrotrachyceratid ammonoids. Such strategies were reported for other coleoids from the German Jurassic: the teuthids *Plesioteuthis* and *Trachyteuthis*^34^ exhibited stomach contents with ammonoid (i.e. lamellaptychi) and actinopterygiid fish remains. Additional evidence for actinopterygiid fish predation on *Phragmoteuthis*^74^ and for predation of coleoids on other coleoids^75^ is provided by the Lower Jurassic Posidonia Shale of Germany. According to its size, abundance and predatory behaviour, *Saurichthys*^5,6^ is possibly the predatory vertebrate in the nektic palaeocommunity of the Polzberg palaeobiota that produced the regurgitalites described herein.

Bromalites contained all size classes from juveniles to adults, entirely preserved or as bitten fragments to shell hash. In other accumulations, crushed ammonoid fragments predominate. The presence of uncrushed or only partly (body chamber) crushed shells has two potential explanations: either the predator swallowed the prey whole or it crushed only the body chamber containing the soft tissues while swallowing. In both cases, the phragmocone remained undamaged. The co-occurrence of the ammonoid genera *Austrotraychyceras* and *Paratrachyceras* along with anaptychi in correlatable numbers suggest that the entire animal was swallowed and became part of bromalites. Accumulations of ammonoid shells consisting of phragmocones have been described in the literature and interpreted as reflecting predation^5,18^. The Polzberg bromalites, both regurgitalites and coprolites, are independent of lithology (argillaceous to limestone) and facies (fine to granular). The trophic and taphonomic features of bromalites, stomach contents and palaeopathologies in ammonoids^34^ have been critically investigated. The conclusion was that coleoids (e.g. *Plesioteuthis* and *Trachyteuthis*) were among the key predators on ammonoids in the Upper Jurassic marine ecosystem^61,66,68,70,71^.

**Figure 7:**
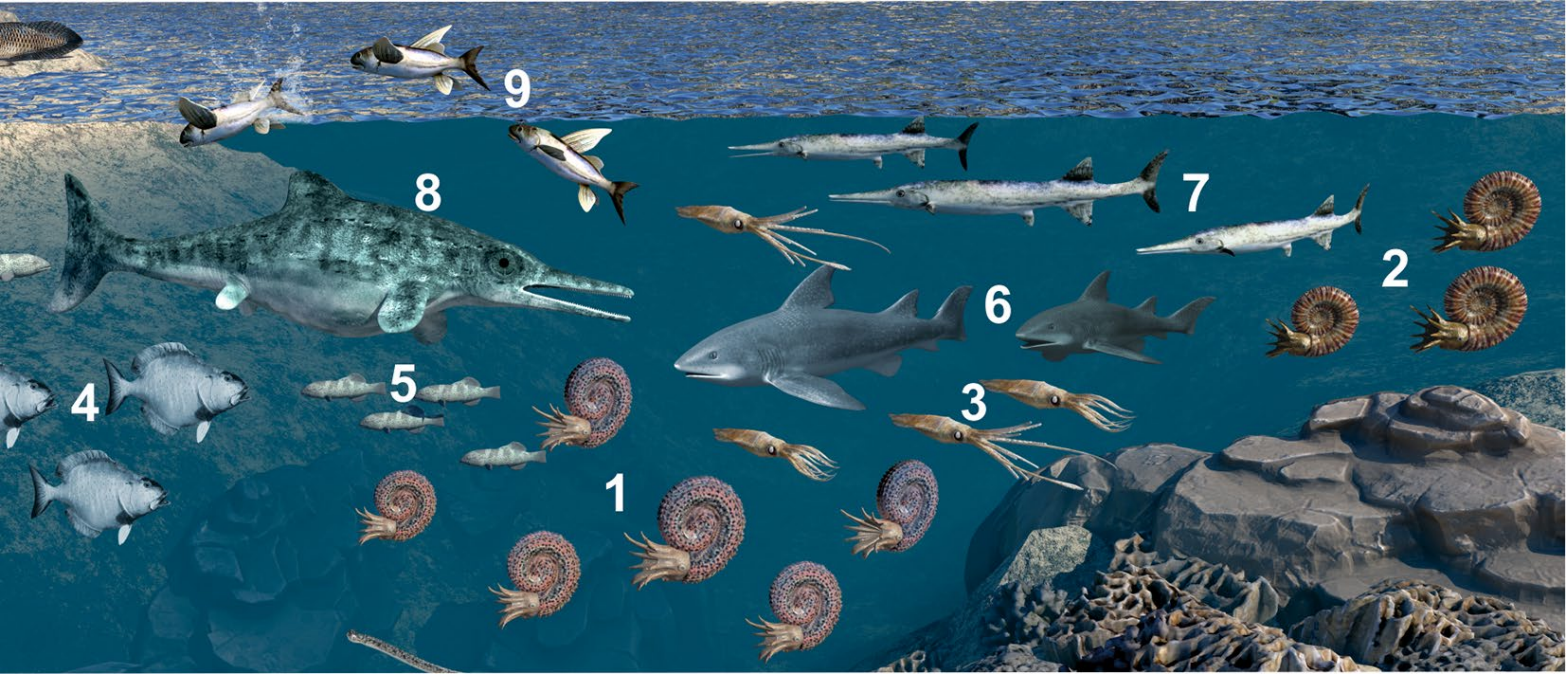
Trophic interactions of the ammonoid members from the Carnian Polzberg palaeobiota. **1** *Austrotrachyceras minor*, **2** *Paratrachyceras haberfellneri*, **3** *Phragmoteuthis bisinuata*, **4** *Polzbergia brochatus*, **5** *Nannolepis elegans*, **6** *Acrodus*, **7** *Saurichthys calcaratus*, **8** predator X, **9** *Thoracopterus niederristi*. Figured members of palaeobiota not to scale. Artwork based on Fig. 5 by AL in Lukeneder and Lukeneder 2021, using CorelDRAW X7; www.coreldraw.com. Final artwork by 7reasons; www.7reasons.net.

Excellent preservation of entire organisms and valuable bromalite findings^5^ serve as documents of trophic interactions (food chains and food web) and predator–prey relationships^6,75,76^. More records of predator–prey relationships from actively swimming organisms are needed: most studies and reports have focused on benthic processes. Nektic members of the Carnian assemblage such as ammonoids (trachyceratids), coleoids (phragmoteuthids) or fish (mostly actinopterygians) form the main constituents of the Polzberg palaeobiota. Their amount, variety and preservation enable conclusions on the palaeo-food web here (Fig. 7)^5^. Bromalites provide evidence for trophic processes and food webs. No sharks have been found at Polzberg^6^ and are therefore currently excluded as possible predators of ammonoids, but reported as directly preying on ammonoids elsewhere, i.e. from Jurassic deposits of France^76^. The shark-like cartilaginous fish *Acrodus*^2,5,6^ is a doubtful taxon from the Polzberg palaeobiota: the specimen described in the literature is apparently lost and no additional remains have been found yet.

## Conclusions

This is the first report on the taphonomy of the ammonoid fauna based on bed-by bed sampling. Our study comprises 3565 ammonoid specimens whose shells are preserved completely or fragmented. The mono- to bispecific ammonoid fauna dominates the Upper Triassic palaeobiota from the Polzberg *Konservat-Lagerstätte*. The Carnian Polzberg locality encompasses all the trophic levels within the marine Polzberg ecosystem including producers, primary and secondary consumers, as well as small and large predators. The deposition of dysoxic sediments of the Reingraben Shales led to the formation of low-oxygen ecosystems here, characterised by laminated deposits. In the overlying oxygenated water column, ceratitid nektic/nektobenthic ammonoids (*Austrotrachyceras*, *Paratrachyceras*), nektic teuthids (*Phragmoteuthis*) and nektic actinopterygiid fish prevailed. The active prey–predator relationships are documented in the variable preservation of ammonoids. The taphonomy and preservational characteristic provide new insights into marine Carnian trophic interactions. We extract a wealth of hidden information on the ammonoid fauna and provide evidence for a preservation of more or less autochthonous deposits here. The presence of fragile nektic and benthic taxa points to special palaeoenvironmental conditions in the Reifling Basin. Triassic invertebrates (e.g., ammonoids, phragmoteuthids, bivalves, gastropods, crustaceans, polychaetes) and vertebrates (actinopterygiids, sarcopterygiids, chondrichtyiids) constituted the marine benthic and nektic communities. Fatal injuries and bromalite contents document the prevailing predator–prey and other synecological relationships among ammonoids and suggests that various cephalopods and fish preyed upon them. The fossil record here contains no evidence for sublethal injuries (e.g. regenerated shells) after the attack, which may reflect the strongly compacted preservation of the shells; we could not detect irregular shell structures or other healed material among this *Konservat-Lagerstätten* material. Bromalites (regurgitalites, coprolites) and ammonoid shell fragment clusters confirm that ammonoid shells were frequently fatally bitten by actinopterygian fish or coleoids. Calm, oxygen-depleted and conditions hostile to scavengers at the sea floor are the prerequisites for excellent preservation of in situ buccal masses with anaptychi and upper jaws within or close to the body chambers of *Austrotraychyceras minor* and *Paratrachyceras haberfellneri*. The low energy (absence of bottom currents) and low oxygenation on the sea floor and in the sediment during the deposition of the Reingraben Shales prevented benthic predators from separating the ammonoid shells from the jaw apparatuses, promoting the extraordinary preservation of the ammonoid conch-jaw association. The dark-coloured organic remains preserved as a black mass in the posterior body chamber are interpreted as muscle remains in the body chambers of the dwarfed ceratitid genera *Austrotrachyceras* and *Paratrachyceras*.

The soft nature of the sediment in the Reifling Basin (i.e. Polzberg subbasin) rules out shell breakage or sublethal to lethal damage on hardgrounds. Predatory pressure was apparently high in this Carnian marine ecosystem, mostly involving agile actinopterygian fish and coleoids feeding on the smaller and slower swimming ammonoids. Redeposition by currents or turbidites can be ruled out based on the quasi-autochthonous character of the deposits. No sorting due to sedimentological or biological effects is evident; no fossil alignments or concentrations were triggered by bottom current transport. Our study confirms the presence of an intact preserved thanatocoenosis.

## Material and methods

3547 recently collected ammonoid remains form the database for the study. They stem from the ravine Polzberggraben (Lunz Nappe, Northern Calcereous Alps) near Polzberg (= Schindelberggraben; or given as Polzberg locality in numerous collections; 4522 additional ammonoid specimens). The ravine is located between mount Föllbaumberg (1014 m) to the west and mount Schindelberg (1066 m) to the east. Overall, the material was collected over the last 140 years (field campaign GBA 1886 and NHMW 1909), with much material provided over the last 10 years by the private collectors Birgitt and Karl Aschauer (both Waidhofen an der Ybbs, Lower Austria), supplemented by findings by the authors during the last 2 years. The fossil remains recorded herein have been investigated with a variety of analytical tools and electronic instruments. The biostratigraphy, systematics and interpretation of facies and palaeoenvironments are based on the summarized data of Lukeneder and Lukeneder^6^. The studied material is housed in the collections of the Natural Museum Vienna (inventory numbers NHMW 2021/0123/0001-3547) and the Geological Survey of Austria (GBA 2021/0170/0001-0138).

Macro-photographs were taken with a Nikon Digital Camera, D 5200 SLR, lens Micro SX SWM MICRO 1:1 Ø52 Nikon AF-S, processed by the free graphic software tool digiCamControl version V.2.1.2.0 at the NHMW. Digital high-quality photomicrographs were taken using a Discovery.V20 Stereo Zeiss microscope. The magnifications were × 10 × 20 and × 40 in incident light mode. Data from the AxioCam MRc5 Zeiss were processed and documented using the AxioVision SE64 Rel. 4.9 imaging system at the NHMW.

Thin sections of rock samples were made in the NHMW laboratories. Samples were embedded in Araldite epoxy resin, sectioned, mounted on the microscope slides and polished with silicon carbide and aluminium oxide powders to a thickness of about 19 μm.

The ammonoid shell composition and internal microstructure were analysed at the laboratories of the Department of Material Sciences and Process Engineering (University of Natural Resources and Life Sciences, Vienna), by SEM imaging on a Quanta™ 250 FEG from FEI (environmental scanning electron microscope with a Shottky field emission source FEG-ESEM) with an EDS tool for microanalysis by energy-dispersive X-ray spectroscopy.

## Data Availability

Raw data related to the fossil material from Polzberg are available from the corresponding author upon request. Measurement data will be made available upon publication in the https://zenodo.org data base and on the project homepage (https://www.nhm-wien.ac.at/forschung/geologie__palaeontologie/forschungsprojekte/polzberg) connected to a server of the Natural History Museum Vienna. Images or additional information are available upon request from Alexander Lukeneder, Natural History Museum Vienna.
